# Specialised palliative care in nursing homes – Retrospective analysis on the basis of claims data

**DOI:** 10.1371/journal.pone.0319001

**Published:** 2025-02-14

**Authors:** Laura Rehner, Kilson Moon, Wolfgang Hoffmann, Neeltje van den Berg

**Affiliations:** 1 Department of Epidemiology of Health Care and Community Health, Institute for Community Medicine, University Medicine Greifswald, Greifswald, Germany; 2 Institute for Nursing Science and Interprofessional Learning, University Medicine Greifswald, Greifswald, Germany; University of Lisbon, Institute of Social and Political Sciences, PORTUGAL

## Abstract

**Background:**

The aim of palliative care is to improve the quality of life of patients with a life limiting illness. In Germany, nursing homes are increasingly the last residence and a common place of death for older people. This renders these institutions as places with a high need for palliative care. However, the frequency of specialised palliative care services in nursing homes in Germany is often low.

**Objectives:**

The aim of this study is 1) to analyse the types and frequencies of services provided by specialised ambulatory palliative care teams in nursing homes in the German federal state of Mecklenburg-Western Pomerania, and 2) to examine whether the frequency of specialised palliative services in nursing homes is comparable to patients living in their private homes.

**Methods:**

The analysis was based on data of the association of statutory health insurance physicians Mecklenburg-Western Pomerania (Germany), data of the statutory health insurance BARMER, and population data. All patients who received specialised ambulatory palliative care in nursing homes in the years 2015–2017 were included in the analysis. For the comparison of the utilisation of specialised ambulatory palliative care in nursing homes with patients in private households, two comparable groups were created using claims, population data and life-year-mortality tables of the general population. It was assumed that people ≥80 years with a life expectancy of <12 months were potential candidates for the utilisation of palliative care. Data were analysed using descriptive statistics and Chi-Square tests.

**Results:**

In Mecklenburg-Western Pomerania, 6,096 patients received specialised ambulatory palliative care in the time period 2015 to 2017. Of these, 16.0% (n = 978) were nursing home residents. The median duration of specialised ambulatory palliative care in nursing homes was 12.0 days, for people in private households 27.2 days. The rate of patients receiving specialised ambulatory palliative care in nursing homes was 4.7%, for people in the comparable group in private households it was 9.2% (p < 0.0001).

**Conclusion:**

Only a small number of nursing home residents received specialised ambulatory palliative care in their last year of life. The rate among those living in their own homes is about twice as high. The results indicate that nursing home residents may have less access to specialised ambulatory palliative care than patients living in private households. Specialised ambulatory palliative care services provision in nursing homes should be improved.

## Background

Palliative care aims to improve the quality of life of patients with a life limiting illness. It attempts to prevent or at least alleviate a patient’s suffering through preventive measures and appropriate care [1]. In Germany, nursing homes are often the last place of residence and a common place of death for older people, which makes these facilities places with a high need for palliative care [2–6]. In the past ten years, the international interest for the implementation of palliative care in nursing homes has grown [3]. Although palliative care has become more common in nursing homes, it is still not routinely available [4,7]. The quality of palliative care in many facilities is rather poor and residents are often hospitalised at the end of their life [4,8,9]. The results of an observational study in Canada among 6000 residents of nursing homes showed that burdensome symptoms and inappropriate end-of-life care had a high prevalence [10]. A randomised study in Australia showed a reduction in hospitalisation among nursing home residents after the structural introduction of specialised palliative care [11]. The same study yielded better quality of death and dying from the perspective of the staff of the nursing homes as well as an increase in staff capability [12]. Pain has a high prevalence in nursing homes [13]. The results of an observational study among 118 nursing homes in Switzerland showed that the presence of structural factors like access to geriatricians, nursing experts, specialised palliative care, and access to pain guidelines were associated to less pain in residents of the nursing homes [14].

People who live in nursing homes usually have more than one chronic condition, and high prevalence of cognitive impairments or dementia. For nurses and physicians, it is often difficult to recognise when a resident is suffering from pain and other symptoms [2]. In some nursing homes, even dying and death are little acknowledged and addressed, which can lead to difficult and undignified dying [8]. A survey in three nursing homes in the USA found that although 69% of the nursing home resident were eligible for palliative care, no resident had received this care [15].

Patients in nursing homes are cared for by nursing staff in most cases. Medical care is provided by external general practitioners (GPs) and medical specialists. Only a few nursing homes have an employed family doctor.

In outpatient healthcare in Germany, both basic and specialised palliative care are offered. Basic outpatient palliative care is provided by GPs and nursing care services. In nursing homes, the nursing staff provides basic palliative care together with external GPs. General palliative care provided by nursing home staff is included in the lump sum for institutional care and therefore not discernible in the reimbursement data [16].

Specialised ambulatory palliative care is provided by specialised multi-professional teams including nurses and physicians [17,18]. To receive services from a specialised ambulatory palliative care team, a prescription from a GP or hospital physician is required. By prescription, these services are also available in nursing homes [18,19]. Specialised ambulatory palliative care is a service that focuses on patients with complex and difficult needs that require a higher level of expertise and skills and more intensive care. It is for patients whose complex medical and nursing needs cannot be adequately covered by basic palliative care provided by the nursing home staff or the GP. Specialised ambulatory palliative care should be available for every eligible patient regardless of diagnosis, age, gender, place of living and financial aspects, including residents of nursing homes [20].

Due to the fact that nursing home residents require more care and suffer more from illness, a higher utilisation of specialised outpatient palliative care is expected in these facilities. This study has two aims: 1) to examine the types of services provided by specialised palliative care teams in nursing homes 2) to determine whether nursing home residents receive more or less specialised ambulatory palliative care compared to patients living in their private households.

## Methods

### Claims data

The analyses were performed on the basis of claims data of the association of statutory health insurance physicians of the Federal State of Mecklenburg-Western Pomerania (Germany), including reimbursement data of all patients with a statutory health insurance. The claims data include anonymised information on demographic parameters (sex, age and postcode of place of residence) and ambulatory care by specialised ambulatory palliative care teams (treatment codes, treatment date and postcode). Data were available from 10 out of 12 specialised ambulatory palliative care teams in Mecklenburg-Western Pomerania. One team does not reimburse via the association of statutory health insurance physicians of Mecklenburg-Western Pomerania, these data are therefore not included in the data set provided. Another team refused to provide data without giving a reason, with the consequence that the association of statutory health insurance physicians was not allowed to provide these data. The data did not contain diagnoses and dates of death. Patients who received services from specialised ambulatory palliative care teams in nursing homes in the years 2015 to 2017 were included in the analysis. In 2017, there were 446 nursing homes in Mecklenburg-Western Pomerania [21].

We received the data of the association of statutory health insurance physicians of the Federal State of Mecklenburg-Western Pomerania at the 9th October 2018. The data were anonymised, the authors had no access to information that could identify individual participants during or after data collection.

Table 1 shows the identified specialised palliative care services provided in the nursing homes with long term care and day care facilities.

**Table 1:** Description of services by specialised ambulatory palliative care teams.

Initial assessment	Evaluation of palliative care needs, development of a therapy concept to alleviate pain and other symptoms, social, psychological and spiritual problems as well as assessment of the domestic and family circumstances. An individualised palliative care plan is drawn up.
Daily lump sum package	All coordinating, advisory and organisational services provided by the specialised ambulatory palliative care team.
Home visit by a nurse	Crisis intervention, guidance and support for primary care providers, evaluation of the care plan, psychosocial care for the patient
Home visit by a physician	Palliative medical measures which, due to their complexity, should be conducted by a physician trained in palliative medicine. These include procedures such as the removal of necroses, port punctures and infusions and pleural punctures.
Active use on-call service (between 20-06 h)	The specialised ambulatory palliative care team was called for crisis intervention between 8 p.m. and 6 a.m. (untimely surcharge).
Complete care specialised ambulatory palliative care	Includes full care of the patient by the specialised ambulatory palliative care team. Full care is provided by nursing and medical members of the specialised ambulatory palliative care team. This service includes symptom control and psychosocial stabilisation, case discussions at least once a week as well as a 24-hour on-call service.
Surcharge travel expenses from 31 km	Surcharge for home visits with a travel distance > 30 km.
Surcharge travel expenses from 31–50. km (Nurse and Physician)	In addition to the surcharge travel expenses from 30st km there is a differentiation from 31st to 50th km and from 51st km. A distinction is further made between home visits by nurses and by physicians.
Surcharge travel expenses from 51 km (Nurse and Physician)	

### Design of the groups: Nursing home residents and people in private households

We used the yearly mortality rates of nursing home residents and people in their private households to build the groups under exposure. The assumption was, that patients with an expected life expectancy of less than 12 months were potential candidates for the utilisation of palliative care. Since the majority of nursing home residents is over 80 years old (72%, Federal Ministry of Health 2021), only this age group was included in both groups.

The yearly mortality rate of nursing home residents ≥80 years was determined on the basis of data from one statutory health insurance (BARMER). These claims data included pseudonymised information on demographic parameters (age, sex, date of death) and the date of nursing home entry. The mortality rate of nursing home residents was calculated using the number of residents ≥80 years old who died within 12 months after entering a nursing home divided by the total number of nursing home residents ≥80 years old. This mortality rate was 35%, so we included 35% of the nursing home residents ≥80 years in the analysis.

To build the comparison group, we included persons of the general population (not living in nursing homes) ≥80 years old. The yearly mortality rates of people ≥80 years, based on three age groups (80–84 years, 85–89 years, ≥90 years) were calculated from the life-year-mortality tables of the general population of the German Statistical Office [22] and projected on the general population of Mecklenburg-Western Pomerania of 2017 [22]. The calculated yearly mortality rate of the general population ≥80 years was 8.9%, so we included 8.9% of the general population ≥80 years of Mecklenburg-Western Pomerania in the comparison group of people in private households.

### Statistical analysis

The statistical analysis was performed using SAS® (Version 9.4, SAS Institute, Care, NC, USA). The descriptive results are reported in percentages, absolute numbers and median with interquartile range (IQR). A Chi-Square test was used to compare the categorical variables. A p-value <  0.05 was considered statistically significant. All methods were performed according to relevant guidelines. The data analysis followed the guidelines of “Good Practice Secondary Data Analysis” [23], Guidelines and recommendations for ensuring Good Epidemiological Practice [24], and the recommendations of STROBE [25].

### Ethics

The study is based on a retrospective analysis on anonymised health care claims data provided by the association of statutory health insurance physicians of Mecklenburg-Western Pomerania. Therefore, no formal ethics committee approval was needed [23]. The approval to use the data without consent of the patients was granted by the supervising authority of the association of statutory health insurance physicians Mecklenburg-Western Pomerania, the Ministry of Economics, Labour and Health of the Federal State of Mecklenburg-Western Pomerania, in accordance with national regulations (§75 (2) of the German Social Security Code V (SGB V)).

## Results

### Patient characteristics and total number of specialised ambulatory palliative care patients in nursing homes

Overall, 6,096 patients in Mecklenburg-Western Pomerania received specialised ambulatory palliative care in the time period 2015 to 2017. The majority of these patients lived in private households (78.5%, n =  4,787); 16.0% (n =  978) lived in nursing homes. The median age of the palliative care patients in nursing homes was 81.5 years, 60.3% (n =  590) were female. The number of patients receiving specialised ambulatory palliative care in nursing homes slightly varies over time, with no increasing or decreasing trend. In 2015, 14.4% (n =  274) of the patients receiving specialised ambulatory palliative care lived in nursing homes, in 2016 18.0% (n =  404), in 2017 14.7% (n =  357). The median duration of specialised ambulatory palliative care in nursing homes was 12.0 days. The median duration of specialised ambulatory palliative care at home was 27.2 days (Table 2).

**Table 2:** Specialised ambulatory palliative care in nursing homes in Mecklenburg-Western Pomerania (2015–2017).

	2015		2016		2017		Total	
	n	%	n	%	n	%	n	%
Total number of patients receiving specialised ambulatory palliative care in Mecklenburg-Western Pomerania	1,904	100.0	2,247	100.0	2,424	100.0	6,096	100.0
Number of patients receiving specialised ambulatory care at home	1,333	70.0	1,623	72.2	1,831	75.5	4,787	78.5
Number of patients receiving specialised ambulatory palliative care in nursing homes, thereof	274^a^	14.4	404^b^	18.0	357	14.7	978	16.0
In stationary care	229	83.6	359	88.9	347	97.2	885	90.5
In partial-stationary care	46	16.8	48	11.9	10	2.8	97	9.9
Age of nursing home residents receiving specialised ambulatory palliative care (years)								
Median (IQR)	81.0 (73.1–86.2)		81.4 (75.5–87.0)		81.5 (74.8–87.4)		81.5 (74.8–86.8)	
Female (%)	58.4		60.9		61.1		60.3	
Duration of specialised ambulatory palliative care in nursing homes (days)								
Median (IQR)	12.5 (4.0–37.0)		13.0 (4.0–34.0)		11.0 (4.0–28)		12.0 (4.0–34.0)	
Duration of specialised ambulatory palliative care at home (days)								
Mean (SD)	26.0 (8.0–66.0)		28.0 (8.0–79.0)		26.0 (8.0–72.0)		27.2 (8.0–72.0)	

^a^One patient received specialised ambulatory palliative care both in a nursing home and in day care.

^b^Three patients received specialised ambulatory palliative care both in nursing home and in day care.

### Provided services of specialised ambulatory palliative care in nursing homes

Table 3 shows the performed specialised palliative care services in nursing homes. To describe the workload of specialised ambulatory palliative care teams, both the number of provided services and the respective number of patients receiving these is presented. Over the years, the number of the service “daily lump sum package” per patient slightly increased from 26.1 on average (95% CI: 22.0; 30.2) in 2015 to 30.9 (95% CI: 25.6; 36.2) in 2017. However, this was statistically not significant. The service “surcharge travel expense from 31st km” (an indication for visits >30 km) was reimbursed on average 7.5 (95% CI: 5.7; 9.4) times per nursing home resident in 2015. The number slightly increased to 11.4 (95% CI: 7.7; 15.0) per nursing home resident in 2017 but this increase was statistically not significant. The surcharge for visits over the 50th km was charged only by nurses of the specialised palliative care team, they visited patients in nursing homes >50 km on average 4.6 (95% CI: 3.2; 5.9) times per nursing home resident in 2015. The number of visits per nursing home resident increased to on average 9.3 (95% CI: 5.3; 13.2) in 2017.

**Table 3:** Performed specialised ambulatory palliative care services in nursing homes in Mecklenburg-Western Pomerania.

Services by specialised ambulatory palliative care teams	2015			2016			2017			Total	
	Services (n)	Patients (n)	Services (n)		Patients (n)	Services (n)		Patients (n)	Services (n)		Patients (n)
Total	11,055	274	16,806		404	16,979		357	44,840		978
Initial assessment	256	248	357		353	294		294	907		892
Daily lump sum package	7,158	274	10,723		401	11,002		356	28,883		974
Home visit (Nurse)	1,841	236	2,752		365	2,749		324	7,342		872
Home visit (Physician)	803	266	1,319		389	1,220		341	3,342		946
Active use on-call service (during 20–06 h)	28	26	53		40	38		33	119		98
Surcharge travel expenses from 31 km	451	60	812		89	876		77	2,139		209
Surcharge travel expenses from 31–50 km											
Nurse	277	33	377		54	317		42	971		122
Physician	144	33	150		56	177		43	441		125
Surcharge travel expenses from 51 km											
Nurse	110	24	263		32	306		33	679		80
Physician	0	0	0		0	0		0	0		0

### Specialised ambulatory palliative care in nursing homes compared to private households

A comparison group of people living in their own households was constructed on the basis of mortality tables of the German statistical office. In 2017, 100,531 people ≥80 years lived in private households in Mecklenburg-Western Pomerania. An analysis of the mortality tables of the German statistical office showed, that the probability of dying of people ≥80 in the next 12 months is 8.9%. In comparison, the probability of dying of the same age group in a nursing home in the next 12 months is 35%.

The percentage of patients ≥80 years of age who received specialised outpatient palliative care in a nursing home was 4.7%. In private households, 820 patients ≥80 years received specialised ambulatory palliative care. On the basis of the assumption described above, the percentage of specialised palliative healthcare in patients living in their own household is 9.2% (Table 4).

**Table 4:** Comparison of specialised ambulatory palliative care by patients in nursing homes with patients in private households based on the assumption, that persons who are likely to die in the next 12 months are potential patients for palliative care (for the year 2017).

	Aged ≥ 80 years	Persons likely to die in the next 12 months		Patients with specialised ambulatory palliative care within 12 months^a^	
	n	n	%	n	%
Nursing home residents	12,293	4,302	35.0%	204	4.7%^c^
Not residents in nursing homes	100,531	8,912^b^	8.9%	820	9.2%^c^

^a^Data from 10 of 12 specialised ambulatory palliative care teams.

^b^Estimated values.

^C^p-value < 0.0001 (with Chi-Square test among person living not and living in nursing homes).

## Discussion

The results show, that only a small number of patients in nursing homes received specialised ambulatory palliative care. The number of visits per patients increased slightly over the three-year observation period, which may indicate both a shift to patients with more severe symptoms or an earlier identification of the need for specialised palliative care. Because the trends are not significant and only cover a few years, further research is needed. Firstly, additional years should be evaluated to see whether the trend is confirmed. Secondly, research on the basis of project or clinical routine data is needed to analyse the cause of the trend.

The daily lump sum package and home visits by nurses of the specialised palliative care team were the most frequently reimbursed services. Nursing home residents with a distance more than 51 km from the place of the specialised ambulatory palliative care team received only visits by nurses of the team. The rate of specialised palliative care in patients in their own homes is twice as high compared to patients in nursing homes with comparable life expectancy. The results suggest that nursing home residents may have less access to specialised ambulatory palliative care than patients living in private households.

A prerequisite for the results to be reliable is the comparability of the groups. Since we have no specific data on the health situation or the specific need for palliative care of the groups, population characteristics must be assessed using parameters from the literature.

Data of a German cohort with n = 633 primary care patients aged 85 and over showed differences in gender distribution between institutionalized (84.7% females) and not institutionalized (65.9% females) patients. Among the non‐institutionalized patients, 12.7% had dementia, among the institutionalized individuals, this was 56.5%. With respect to other health‐related factors (visual impairment, hearing impairment, depressive symptoms, level of frailty, number of chronic illnesses), patients in nursing homes showed poorer health compared to not institutionalized patients [26].

An important indicator of health status is the presence of a formal level of care. Care levels 1 to 5 form a scale that reflects the degree of independence and impairment of the person in need of care. The higher the care level, the greater the need for help. Because the formal level of care is measured in a standardized manner and is a good measure of the health situation, it would be desirable to supplement the data with the level of care for subsequent studies. To live in a nursing home, at least a formal level of care of 2 is required. In the general population, 44.3% of women and 26.9% of men aged 80 and over have a formal level of care [27]. These data show, that the health situation of patients in nursing homes is not comparable to patients living in their own household.

it is important to notice, that we don’t assume that patients in nursing homes need more specialised palliative care than patients living in private households, but we don’t expect them to need less care either [28,29]. Hence the results indicate that there is an undersupply of specialised ambulatory palliative care in nursing homes.

Since the survival time of patients from nursing home admission is about 1.9 years on average, one of the main tasks of these institutions it to accompany patients in the terminal phase [4,30]. Specialised ambulatory palliative care is needed whenever there is a complex palliative care situation and the needs of the patients can no longer be covered by the usual care provided in the nursing home [20]. One reason for the low uptake of specialised ambulatory palliative care may be that the patients’ needs are not being fully recognised. There are no measures for the quality of palliative care in the nursing homes in the study region included in the data. However, we performed a survey among all providers of palliative care in the study region, including GPs with and without additional training in palliative care, specialised palliative care teams, nursing homes, outpatient nursing care services, and palliative care wards in hospitals. Main results of the survey with respect to nursing homes were insufficient knowledge and quality of palliative care [9]. Furthermore, other studies show, that the knowledge of nurses in nursing homes about palliative care is not sufficient and should be improved [30,31]. A lack of medical knowledge among nurses and an insufficient proportion of nurses with a qualification in palliative care as well as a predominant share of less qualified nursing assistance in the personnel key could explain that palliative care needs are not recognised or not appropriately covered in nursing homes [31].

Residents in the terminal phase are more time-consuming and require more work. Time pressure, the conflict between wanting to care properly for the dying resident but not being able to care for each individual resident sufficiently makes it difficult to perform general palliative care in nursing homes. This kind of moral distress to provide adequate care for the residents is a phenomenon which can affect physical wellbeing of nursing home staff [32]. Furthermore, nursing home residents in Germany are often unnecessarily hospitalised in terminal disease stages [30]. Hoffmann and Allers [33] found that of the 67,328 nursing home residents identified, 74.3% were hospitalised at least once in the last 12 months of life. In the last 28 days of life, 51.5% were hospitalised [32]. These admissions of nursing home residents could be prevented with advanced care planning and early palliative care consultations [30].

Another aspect could be that some GPs (caring for both nursing home residents and patients in their own homes) may have insufficient knowledge about palliative care to identify the need for specialised care. Although there is a support program with additional training for GPs available, all GPs can bill for basic palliative services. The number of basic palliative services varies greatly regionally, Mecklenburg-Wester Pomerania is about average [34].

In 2019, we identified 139 GPs with additional training in palliative medicine in the study region. In 2024, there were 150 GPs with this training. This slight increase in the number of GPs with additional palliative training may indicate that this training is not attractive enough with respect to financial benefits or time resources.

The number of basic palliative services is decreasing throughout Germany (measured between 2016–2019), which could be due to a lack of resources [34].

GPs might also be concerned to lose their care mandate to palliative care specialists as well as about the comparatively higher level of bureaucracy for the prescription of specialised ambulatory palliative care [35].

One more reason for the low utilisation of services could be that the specialised ambulatory palliative care teams do not have enough capacity to care for nursing home residents as needed. We explored the size of the specialised palliative care teams in 2019. The size of the overall team ranged from 4 to 33 persons. The number of nurses in the teams ranged from 3 to 22, the number of physicians from 1 to 15. In small teams, it is very difficult to guarantee constant availability.

In a survey of providers involved in palliative care in the study region, a lack of doctors and nurses with additional training in palliative medicine was mentioned most frequently as an aspect that endangers adequate palliative care. Furthermore, the results indicate problems in access to palliative care in rural areas [9].

In Germany, the size of organizations for specialised ambulatory palliative care teams varies a lot between 1 and 298 staff members. Some of the organizations do not employ their own physicians and nurses but work with cooperating practices and nursing services [36]. Therefore, the capacity of specialised ambulatory palliative care teams varies greatly between regions [34]. Small teams will therefore carefully assess the indications for care in combination with other parameters (e.g., living setting, distance) and possibly reject patients in nursing homes who are supposedly better cared for.

In 2015, the Hospice and Palliative Care Act was passed in Germany [37]. The aim of this law is to improve hospice and palliative care in Germany [34]. A new paragraph “Health Care Planning for the Final Phase of Life” (§132) was added to Social Code Book V. It is based on the concept of advanced care planning. The aim is to create an individual care plan for the end-of-life phase for nursing home residents. The purpose is to reduce unnecessary and, above all, unwanted emergency medical interventions and hospital admissions. Furthermore, the number of non-indicated and unwanted therapies at the end of life should be reduced. In nursing homes, possible support and services for end-of-life care ought to be offered as well as information about medical procedures during the terminal phase [38]. The results indicate that the amount of provided care increased. However, the proportion of nursing home residents receiving specialised ambulatory palliative care did not show an increasing trend. Still, the results show that relatively few services of specialised outpatient palliative care were utilised compared to private households.

The analysis was performed on the basis of data from the federal state of Mecklenburg-Western Pomerania. The number of both basic and specialised palliative care services is average compared to the other German federal states [34].

A survey among nurses in nursing homes in Germany in the year 2022 showed, that the use of specialised palliative care teams in nursing homes is rather rare. About half of the nurses surveyed said that cooperation with specialised palliative care teams rarely or never exists [7]. We therefore assume that the results of our study are transferable to other regions in Germany. In order to provide needs-based care in nursing homes, to prevent unnecessary hospital admissions in the terminal phase and to protect the nursing home staff from psychological burden, the staff-to-resident ratio should be increased and nurses with palliative care knowledge should be available in all nursing homes [32,39].

### Limitations and strengths

The analysis has some limitations. A limitation is, that the used data were not collected for the purpose to evaluate palliative care but to reimburse healthcare services. The data represent money flows in the healthcare system and has limitations with respect to actual healthcare services provision. Another limitation regarding the reimbursement data is the age of the data. Having said that, the care system did not change with regard to the question addressed in this analysis. Additionally, the results of a recent analysis, in which we collected data from patient files in nursing homes, show a very low utilization of SAPV services, so that we can assume that the problem described here is still current (data not yet published).

It is not possible to make a general statement about specialised ambulatory palliative care in Mecklenburg-Western Pomerania due to missing data of two specialised palliative care teams. Due to these missing data, there is probably an underestimation of the utilization of specialised ambulatory palliative care in their respective regions. However, this is the case both in nursing homes and in private households. We therefore expect that these missing data do not affect the results of the comparison between the groups.

The data did not include diagnoses. However, the validity of the diagnoses in reimbursement data, especially in outpatient care, is also limited, because of lack of standardization of diagnostics, different points of time of diagnostics and the purpose of documentation (reimbursement). The dataset also did not contain formal levels of care, and only included ambulatory care provided by specialised ambulatory palliative care teams.

One limitation concerns the comparison group. It was constructed of patients ≥80 years with an expected survival of <12 months and projected the specialised palliative care service into this group. However, since it can be assumed that the group of patients living in their own homes is healthier than the patients in nursing homes, the control group was conservatively chosen.

A strength of the analysis is that data on the place of the performance of the services was available. A further strength is the availability of data for all patients, independent of their health insurance provider.

The results of the analysis indicate that nursing home residents may have less access to specialised ambulatory palliative care than patients with a comparable life expectancy living in private households.

Since general palliative care by nurses is included in the lump sum for care in nursing homes and not reported separately, it is not possible to assess the amount of general palliative care in nursing homes. In some nursing homes with a good concept for palliative care, there may be some compensation for more specialised palliative care. However, palliative care provided by nursing home staff cannot fully address the complex medical and nursing needs of patients.

Improvement of palliative care skills and knowledge about palliative care among nurses in nursing homes and GPs could lead to more specialised palliative care in nursing homes. A possible solution could be that nursing homes receive unbureaucratic support from specialised palliative care teams via telephone or videoconference. This would likely reduce the workload of both the nursing home staff and the specialised ambulatory palliative care teams in these facilities and provide more adequate care for the residents in the last phase of life. A collaborative approach could prevent emotional stress for carers and relatives and prevent the nursing home residents from unnecessary suffering.

On the basis of the results of the presented analysis, we developed a cluster randomized study with ten nursing homes. The nursing homes of the intervention group implemented a qualification for basic palliative care. The aim was to improve nursing skills in order to identify the need for palliative care. The results of the study (publication in preparation) will serve as basis for the development of telemedicine concepts to enable low-threshold support from specialised palliative care teams or specialised palliative care wards in hospitals.

## Conclusions

The results of the analysis show, that specialised palliative care may be insufficient in nursing homes. However, nursing homes are complex institutions, more knowledge is needed about how to improve palliative care in these facilities and how the cooperation between nursing homes and specialised ambulatory palliative care teams can be improved.

## References

[1] World Health Organization. WHO Definition of Palliativ Care2. 2002 [cited 2022 Sep 30]. Available from: https://www.dgpalliativmedizin.de/images/stories/WHO_Definition_2002_Palliative_Care_englisch-deutsch.pdf

[2] Collingridge Moore D, Payne S, Van den Block L, Ling J, Froggatt K; PACE. Strategies for the implementation of palliative care education and organizational interventions in long-term care facilities: a scoping review. Palliat Med. 2020;34(5):558–70. doi: 10.1177/0269216319893635 32009516 PMC7222696

[3] Froggatt K, Payne S, Morbey H, Edwards M, Finne-Soveri H, Gambassi G, et al; PACE. Palliative care development in European care homes and nursing homes: application of a typology of implementation. J Am Med Dir Assoc. 2017;18(6):550.e7–14. doi: 10.1016/j.jamda.2017.02.016 28412166 PMC5754324

[4] Allers K, Hoffmann F, Schnakenberg R. Hospitalizations of nursing home residents at the end of life: a systematic review. Palliat Med. 2019;33(10):1282–98. doi: 10.1177/0269216319866648 31368855 PMC6899437

[5] Hall S, Kolliakou A, Petkova H, Froggatt K, Higginson IJ. Interventions for improving palliative care for older people living in nursing care homes. Cochrane Database Syst Rev. 2011;2011(3):CD007132. doi: 10.1002/14651858.CD007132.pub2 21412898 PMC6494579

[6] Dasch B, Zahn PK. Place of death trends and utilization of outpatient palliative care at the end of life—analysis of death certificates (2001, 2011, 2017) and pseudonymized data from selected palliative medicine consultation services (2017) in Westphalia, Germany. Dtsch Arztebl Int. 2021;118(19):331–8. doi: 10.3238/arztebl.m2021.0124 34180794 PMC8336643

[7] Behrendt S, Argüello Guerra F, Räker M, Jürchott K, Klauber J, Schwinger A. Die Versorgung von Pflegeheimbewohnenden am Lebensende aus Sicht der Pflege. Eine Befragung in deutschen Pflegeheimen. [cited 2024 Nov 26]. Available from: https://repository.publisso.de/resource/frl:6434217/data

[8] Bükki J, Neuhaus PM, Paal P. End of life care in nursing homes: Translating focus group findings into action. Geriatr Nurs. 2016;37(6):440–5. doi: 10.1016/j.gerinurse.2016.06.011 27406626

[9] Rehner L, Moon K, Hoffmann W, van den Berg N. Die allgemeine und spezialisierte Palliativversorgung aus Sicht von Leistungserbringern der Hospiz- und Palliativversorgung – eine Querschnittserhebung. Gesundheitswesen. 2021;83(12):986–92. 33302320 10.1055/a-1276-0391

[10] Hoben M, Chamberlain SA, Knopp-Sihota JA, Poss JW, Thompson GN, Estabrooks CA. Impact of symptoms and care practices on nursing home residents at the end of life: a rating by front-line care providers. J Am Med Dir Assoc. 2016;17(2):155–61. doi: 10.1016/j.jamda.2015.11.002 26706418

[11] Forbat L, Liu WM, Koerner J, Lam L, Samara J, Chapman M, et al. Reducing time in acute hospitals: a stepped-wedge randomised control trial of a specialist palliative care intervention in residential care homes. Palliat Med. 2020;34(5):571–9. doi: 10.1177/0269216319891077 31894731

[12] Liu WM, Koerner J, Lam L, Johnston N, Samara J, Chapman M, et al. Improved quality of death and dying in care homes: a palliative care stepped wedge randomized control trial in Australia. J Am Geriatr Soc. 2020;68(2):305–12. doi: 10.1111/jgs.16192 31681981

[13] Lukas A, Mayer B, Fialová D, Topinkova E, Gindin J, Onder G, et al. Treatment of pain in European nursing homes: results from the Services and Health for Elderly in Long TERm Care (SHELTER) study. J Am Med Dir Assoc. 2013;14(11):821–31. doi: 10.1016/j.jamda.2013.04.009 23746948

[14] Baumgartner-Violand S, Brunkert T, Cassidy S, Blatter C, Favez L, Zúñiga F. Association between modifiable structural and process factors and the quality indicator pain in nursing home residents: a multicentre cross-sectional survey. J Adv Nurs. 2024. doi: 10.1111/jan.16567 39441541 PMC12159391

[15] Stephens CE, Hunt LJ, Bui N, Halifax E, Ritchie CS, Lee SJ. Palliative care eligibility, symptom burden, and quality-of-life ratings in nursing home residents. JAMA Intern Med. 2018;178(1):141–2. doi: 10.1001/jamainternmed.2017.6299 29159368 PMC5833507

[16] Gemeinsamer Bundesausschuss (G-BA). Richtlinie des Gemeinsamen Bundesausschusses über die Verordnung von häuslicher Krankenpflege (Häusliche Krankenpflege-Richtlinie). 2020 [cited 2020 Nov 11]. Available from: https://www.g-ba.de/downloads/62-492-2261/HKP-RL_2020-09-17_iK-2020-10-01.pdf

[17] Cremer-Schaeffer P, Radbruch L. Palliativversorgung im Blickwinkel gesetzlicher und regulatorischer Vorgaben in Deutschland. Bundesgesundheitsblatt - Gesundheitsforschung - Gesundheitsschutz. 2012;55(2):231–7. doi: 10.1007/s00103-011-1408-9 22290167

[18] Freytag A, Krause M, Bauer A, Ditscheid B, Jansky M, Krauss S, et al; SAVOIR Study group. Study protocol for a multi-methods study: SAVOIR - evaluation of specialized outpatient palliative care (SAPV) in Germany: outcomes, interactions, regional differences. BMC Palliat Care. 2019;18(1):12. doi: 10.1186/s12904-019-0398-5 30684958 PMC6348077

[19] Radbruch L, Payne S. Standards und Richtlinien für Hospiz- und Palliativversorgung in Europa: Teil 2. Zeitschrift für Palliativmedizin. 2011;12(06):260–70. doi: 10.1055/s-0031-1276957

[20] Radbruch L, Payne S. Standards und Richtlinien für Hospiz- und Palliativversorgung in Europa: Teil 1. Zeitschrift für Palliativmedizin. 2011;12(05):216–27. doi: 10.1055/s-0031-1276909

[21] Statistisches Bundesamt Destatis. Pflegestatistik - Pflege im Rahmen der Pflegeversicherung 2017, in Ländervergleich - Pflegeheime. 2018 [cited 2025 Jan 29]. Available from: https://www.statistischebibliothek.de/mir/receive/DEHeft_mods_00096708

[22] Statistisches Bundesamt Destatis, Sterbetafel 2017/2019. 2020 [cited 2025 Jan 29]. Available from: https://www.statistischebibliothek.de/mir/receive/DEHeft_mods_00131507

[23] Swart E, Gothe H, Geyer S, Jaunzeme J, Maier B, Grobe TG, et al. Gute Praxis Sekundärdatenanalyse (GPS): Leitlinien und Empfehlungen. Gesundheitswesen. 2015;77(2):120–6. doi: 10.1055/s-0034-1396815 25622207

[24] Hoffmann W, Latza U, Baumeister SE, Brünger M, Buttmann-Schweiger N, Hardt J, et al. Guidelines and recommendations for ensuring Good Epidemiological Practice (GEP): a guideline developed by the German Society for Epidemiology. Eur J Epidemiol. 2019;34(3):301–17. doi: 10.1007/s10654-019-00500-x 30830562 PMC6447506

[25] The EQUATOR Network. STROBE guidelines for scientific reporting on observational studies. [cited 2021 Jul 16] Available from: https://www.equator-network.org/reporting-guidelines/strobe/

[26] Hajek A, Luppa M, Brettschneider C, van der Leeden C, van den Bussche H, Oey A, et al. Correlates of institutionalization among the oldest old-Evidence from the multicenter AgeCoDe-AgeQualiDe study. Int J Geriatr Psychiatry. 2021;36(7):1095–102. doi: 10.1002/gps.5548 33772875

[27] Gaertner B, Scheidt-Nave C, Koschollek C, Fuchs J. Gesundheitliche Lage älterer und hochaltriger Menschen in Deutschland: Ergebnisse der Studie Gesundheit 65+. J Health Monit. 2023;8(3):7–29. doi: 10.25646/11663 37829119 PMC10565703

[28] Amblàs-Novellas J, Murray SA, Espaulella J, Martori JC, Oller R, Martinez-Muñoz M, et al. Identifying patients with advanced chronic conditions for a progressive palliative care approach: a cross-sectional study of prognostic indicators related to end-of-life trajectories. BMJ Open. 2016;6(9):e012340. doi: 10.1136/bmjopen-2016-012340 27645556 PMC5030552

[29] Esteban-Burgos AA, Lozano-Terrón MJ, Puente-Fernandez D, Hueso-Montoro C, Montoya-Juárez R, García-Caro MP. A New Approach to the Identification of Palliative Care Needs and Advanced Chronic Patients among Nursing Home Residents. Int J Environ Res Public Health. 2021;18(6):3171. doi: 10.3390/ijerph18063171 33808567 PMC8003433

[30] Allers K, Hoffmann F. Mortality and hospitalization at the end of life in newly admitted nursing home residents with and without dementia. Soc Psychiatry Psychiatr Epidemiol. 2018;53(8):833–9. doi: 10.1007/s00127-018-1523-0 29721593

[31] Smets T, Pivodic L, Piers R, Pasman HRW, Engels Y, Szczerbińska K, et al. The palliative care knowledge of nursing home staff: The EU FP7 PACE cross-sectional survey in 322 nursing homes in six European countries. Palliat Med. 2018;32(9):1487–97. doi: 10.1177/0269216318785295 29972343 PMC6158686

[32] Midtbust MH, Alnes RE, Gjengedal E, Lykkeslet E. Perceived barriers and facilitators in providing palliative care for people with severe dementia: the healthcare professionals’ experiences. BMC Health Serv Res. 2018;18(1):709. doi: 10.1186/s12913-018-3515-x 30208872 PMC6134769

[33] Hoffmann F, Allers K. Krankenhausaufenthalte von Pflegeheimbewohnern in der letzten Lebensphase: eine Analyse von Krankenkassenroutinedaten. Z Gerontol Geriatr. 2021;54(3):247–54. doi: 10.1007/s00391-020-01716-3 32185465 PMC8096747

[34] Ditscheid B, Meissner F, Gebel C, Hennig B, Marschall U, Meißner W, et al. Inanspruchnahme von Palliativversorgung am Lebensende in Deutschland: zeitlicher Verlauf (2016–2019) und regionale Variabilität. Bundesgesundheitsblatt Gesundheitsforschung Gesundheitsschutz. 2023;66(4):432–42. doi: 10.1007/s00103-023-03683-7 36897332 PMC10063517

[35] Peter S, Volkert AM, Pfaff H, Radbruch L, Rolke R, Voltz R, et al. General practitioners’ perspectives on general and specialized palliative home care in North Rhine, Germany: an explorative focus group study. Am J Hosp Palliat Care. 2021;38(1):32–8. doi: 10.1177/1049909120920541 32342700

[36] Jansky M, Heyl L, Hach M, Kranz S, Lehmann T, Freytag A, et al. Structural characteristics and contractual terms of specialist palliative homecare in Germany. BMC Palliat Care. 2023;22(1):166. doi: 10.1186/s12904-023-01274-6 37904160 PMC10617175

[37] Gesetz zur Verbesserung der Hospiz- und Palliativversorgung in Deutschland (Hospiz- und Palliativgesetz – HPG). Bundesgesetzblatt. 2015;48:2114–8.

[38] Melching H. Neue gesetzliche Regelungen fur die Palliativversorgung und ihre Implikationen fur Politik und Praxis. Bundesgesundheitsblatt Gesundheitsforschung Gesundheitsschutz. 2017;60(1):4–10. doi: 10.1007/s00103-016-2480-y 27995269

[39] Allers K, Fassmer AM, Spreckelsen O, Hoffmann F. End-of-life care of nursing home residents: A survey among general practitioners in northwestern Germany. Geriatr Gerontol Int. 2020;20(1):25–30. doi: 10.1111/ggi.13809 31760683

